# Metabolite profile of Bambara groundnut (*Vigna subterranea*) and *dawadawa* (an African fermented condiment) investigation using gas chromatography high resolution time-of-flight mass spectrometry (GC-HRTOF-MS)

**DOI:** 10.1016/j.heliyon.2021.e06666

**Published:** 2021-04-03

**Authors:** Janet Adeyinka Adebiyi, Patrick Berka Njobeh, Oluwafemi Ayodeji Adebo, Eugenie Kayitesi

**Affiliations:** a Department of Biotechnology and Food Technology, Faculty of Science, University of Johannesburg, PO Box 17011, Doornfontein Campus, Gauteng, South Africa; b Department of Consumer and Food Sciences, Faculty of Natural and Agricultural Sciences, University of Pretoria, Private Bag X20, Hatfield 0028, Pretoria, South Africa

**Keywords:** Fermented condiment, GC-HRTOF-MS, Legume, Metabolites, Profiling

## Abstract

Metabolite profile provides an overview and avenue for the detection of a vast number of metabolites in food sample at a particular time. Gas chromatography high resolution time-of-flight mass spectrometry (GC-HRTOF-MS) is one of such techniques that can be utilized for profiling known and unknown compounds in a food sample. In this study, the metabolite profiles of Bambara groundnut and *dawadawa* (unhulled and dehulled) were investigated using GC-HRTOF-MS. The presence of varying groups of metabolites, including aldehydes, sterols, ketones, alcohols, nitrogen-containing compounds, furans, pyridines, acids, vitamins, fatty acids, sulphur-related compounds, esters, terpenes and terpenoids were reported. Bambara groundnut fermented into derived *dawadawa* products induced either an increase or decrease as well as the formation of some metabolites. The major compounds (with their peak area percentages) identified in Bambara groundnut were furfuryl ether (9.31%), bis (2-(dimethylamino)ethyl) ether (7.95%), 2-monopalmitin (7.88%), hexadecanoic acid, methyl ester (6.98%), 9,12-octadecadienoic acid (Z,Z) and 2-hydroxy-1-(hydroxymethyl)ethyl ester (5.82%). For dehulled *dawadawa*, the significant compounds were palmitic acid, ethyl ester (17.7%), lauric acid, ethyl ester (10.2%), carbonic acid, 2-dimethylaminoethyl 2-methoxyethyl ester (7.3%), 9,12-octadecadienoic acid (Z,Z)-, 2-hydroxy-1-(hydroxymethyl)ethyl ester (5.13%) and maltol (4%), while for undehulled *dawadawa*, it was indoline, 2-(hydroxydiphenylmethyl) (26.1%), benzoic acid, 4-amino-4-hydroximino-2,2,6,6-tetramethyl-1-piperidinyl ester (8.2%), 2-undecen-4-ol (4.7%), 2-methylbutyl propanoate (4.7%) and ë-tocopherol (4.3%). These observed metabolites reported herein provides an overview of the metabolites in these investigated foods, some of which could be related to nutrition, bioactivity as well as sensory properties. It is important to emphasize that based on some of the metabolites detected, it could be suggested that Bambara groundnut and derived *dawadawa* might serve as functional foods that are beneficial to health.

## 1 Introduction

Fermentation is a biochemical process that results in modifications (increase/decrease) as well as formation (synthesis) of metabolites. These metabolites contribute to the nutritional qualities, taste, shelf life, safety, aroma, health promoting properties and overall composition of fermented foods. Traditionally, these fermented foods are produced from legumes or cereals and undergo various forms of fermentation, such as alkali fermentation, lactic acid fermentation, acetic acid fermentation and alcoholic fermentation (Oliveira et al., 2014). The fermentation process whereby the pH of a legume increases to alkaline levels and possibly pH values of 9 and above is known as alkaline fermentation (Omafuvbe et al., 2002) and such is due to breakdown of proteins to ammonia, peptides and amino acids (Wang and Fung, 1996; Kiers et al., 2000).

In African and Asian countries, several alkaline fermented food condiments, such as *dawadawa, thua-nao*, *iru*, *natto* and *soumbala,* are mostly produced from legumes and from Bambara groundnut (Fadahunsi and Olubunmi, 2009; Parkouda et al., 2009; Ademiluyi and Oboh, 2011; Akanni et al., 2018a; Muhammad et al., 2018). These fermented condiments are processed mostly through natural fermentation; however, this is not always the case, as some undergo controlled fermentation. During the production of *dawadawa*, raw legume seeds are soaked, manually dehulled and boiled to soften the seeds. The boiled softened raw seeds are wrapped with leaves (such as banana leaves), kept in sacks or bags and incubated in a plastic bowl/calabash/earthen pot for three to five days (the fermentation period is usually based on human discretion) (Onawola et al., 2012). The most important and major processing step for this product is fermentation and has been proven to enhance the organoleptic and beneficial health properties of fermented legumes (Oboh et al., 2009; Ademiluyi and Oboh, 2011; Chinma et al., 2020).

Monitoring of these metabolic, biochemical, physicochemical and structural changes occurring during the fermentation process may be somewhat difficult, necessitating the utilization of techniques and robust equipment, which can provide a better overview of these metabolites. Gas chromatography–mass spectrometry is a non-biased, comprehensive and sensitive technological system used for the detection of diverse volatile and semi-volatile metabolites (Adebo et al., 2021). It has advantages of better resolution, high sensitivity, good reproducibility and, with the necessary databases, makes identification of compounds relatively easier (Hu et al., 2018). Particularly for gas chromatography coupled with high-resolution time of flight mass spectrometry, it is a powerful and highly effective analytical tool with excellent capabilities including a better chromatographic separating capability over a wide mass range with an accurate mass measurement (Brits et al., 2018; Kewuyemi et al., 2020). The exact mass information and mass resolution provided by high-resolution time-of-flight mass spectrometry (HR-TOFMS) can enhance target identification of compound and also assist in the identification of unknown compounds (Ubukata et al., 2015).

While few authors have studied the composition of *dawadawa* (Akanni et al., 2018a; Onyenekwe et al., 2012; Adebiyi et al., 2019), there is still no study providing a comparison of the metabolite profile of two types of *dawadawa* (dehulled and unhulled) from Bambara groundnut (BGN) obtained through natural fermentation. Thus, this study was aimed to profile metabolites in BGN and *dawadawa* (dehulled and unhulled) using GC-HRTOF-MS, envisaging that the metabolites would be beneficial to consumers of these products.

## 2 Materials and methods

### 2.1 Raw materials and sample preparation

Bambara groundnuts (mixed varieties) (i.e. brown, cream and red) used in this study were procured from a local farmer in Limpopo Province, South Africa. These were subsequently sorted to remove extraneous material or debris and cleaned with water.

### 2.2 Fermentation of Bambara groundnut into *dawadawa*

The production of both dehulled and unhulled *dawadawa* has been previously described in our earlier study Adebiyi et al. (2019). Briefly, the BGN was soaked for 24 h in water and dehulled manually, the dehulled raw seeds were rinsed with water and boiled for 1 h. Later, the boiled and cooled BGN seeds were spread and covered with a sterile banana leaves, wrapped in jute bags and incubated at 35 °C for 84 h. For the unhulled *dawadawa* (UHD), the hulls or seed coats were not removed after soaking and were incubated at 35 °C for 120 h. The fermentation time and temperature conditions (35 °C for 84 h and 35 °C for 120 h, for DD and UHD, respectively) that were used for producing the *dawadawa* samples was guided by the optimized results earlier achieved and reported in Adebiyi et al. (2020). The samples were freeze-dried at −55 °C for 24 h (LyoQuest Telstar Technologies, Spain) and stored at 4 °C prior to analysis.

### 2.3 Sample preparation for metabolite profiling

At the initial stage of sample preparation, different extraction solvents (all analytical grade) [100% acetonitrile (ACN), 100% methanol (MeOH), 100% water (H_2_O), 80% ACN in H_2_O, 80% MeOH in H_2_O, 50% ACN:MeOH (v/v), 1% HCl in MeOH, ACN:MeOH:H_2_O (4,4,2, v/v/v) and isopropanol:ACN:H2O (4,4,2, v/v/v)] were used to investigate for a possible range of available metabolites in the samples.

An informed compromise was reached and extraction using 80% MeOH in H_2_O was finally adopted based on a wider range of relevant metabolites detected on the GC-HRTOF-MS system. Briefly, 10 mL of 80% MeOH was added to 1 g of freeze-dried sample, agitated and the mixture sonicated in an ultrasonic bath (Integral Systems Ultrasonic Bath UMC 5, Labotec, South Africa) at 4 °C for 1 h. The mixture was then centrifuged at 3 500 rpm for 5 min at 4 °C (Eppendorf 5702R, Merck South Africa), transferred into a 250 mL round bottom flask and concentrated at 30 °C, under pressure using a rotary evaporator (Buchi, Switzerland). The extract was reconstituted with 1 mL chromatographic grade MeOH (Sigma Aldrich, Germany) and filtered into a dark amber vial for analysis. The extraction was done in triplicates for each sample.

### 2.4 GC-HRTOF-MS analysis

A mass calibration of the instrument was performed prior to analysis on the LECO Pegasus GC-HRTOF-MS system (LECO Corporation, St Joseph, MI, USA) and subsequent sample analyses done using the method of Adebo et al. (2019). The samples were then analyzed on the GC-HRTOF-MS system equipped with an Agilent 7890A (Agilent Technologies, Inc., Wilmington, DE, USA) gas chromatograph running in a high-resolution. This was coupled to a Gerstel MPS multipurpose autosampler (Gerstel Inc. Germany) and analytical column was a Rxi®-5ms (30 m × 0.25 mm ID × 0.25 μm) (Restek, Bellefonte, USA). One microlitre (μL) of each sample was injected (in a splitless mode) with helium as the carrier gas at a constant flow rate (1 mL/min). The transfer line and inlet temperatures were 225 °C and 250 °C respectively. The oven temperature was initially set at 70 °C, held for 0.5 min, ramped at 10 °C/min to 150 °C and held for 2 min. The oven temperature was later ramped at 10 °C/min to 330 °C and held for 3 min Triplicate extraction for each sample were respectively injected once into the GC-HR-TOF-MS equipment as well as solvent blanks to observe impurities and possible contamination.

### 2.5 Data analysis

From the data obtained, peak picking, retention time alignment, peak matching and detection were done on the ChromaTOF-HRT® software (LECO Corporation, St Joseph, MI, USA). Other data processing parameters adopted included a signal to noise ratio of 100 and a minimum match similarity of >70% prior to when compound name is assigned, using the Mainlib, Feihn and NIST metabolomics database by comparing the molecular formula, retention time and mass spectra data. Percentage peak areas were subsequently calculated, and the respective observed *m/z* fragments obtained from the ChromaTOF-HRT® data station were recorded after which the metabolite class was annotated with corresponding *m/z* fragments and molecular formula.

## 3 Results and discussion

### 3.1 Metabolites of Bambara groundnut and *dawadawa* profiled using GC-HRTOF-MS

**Figure 1:**
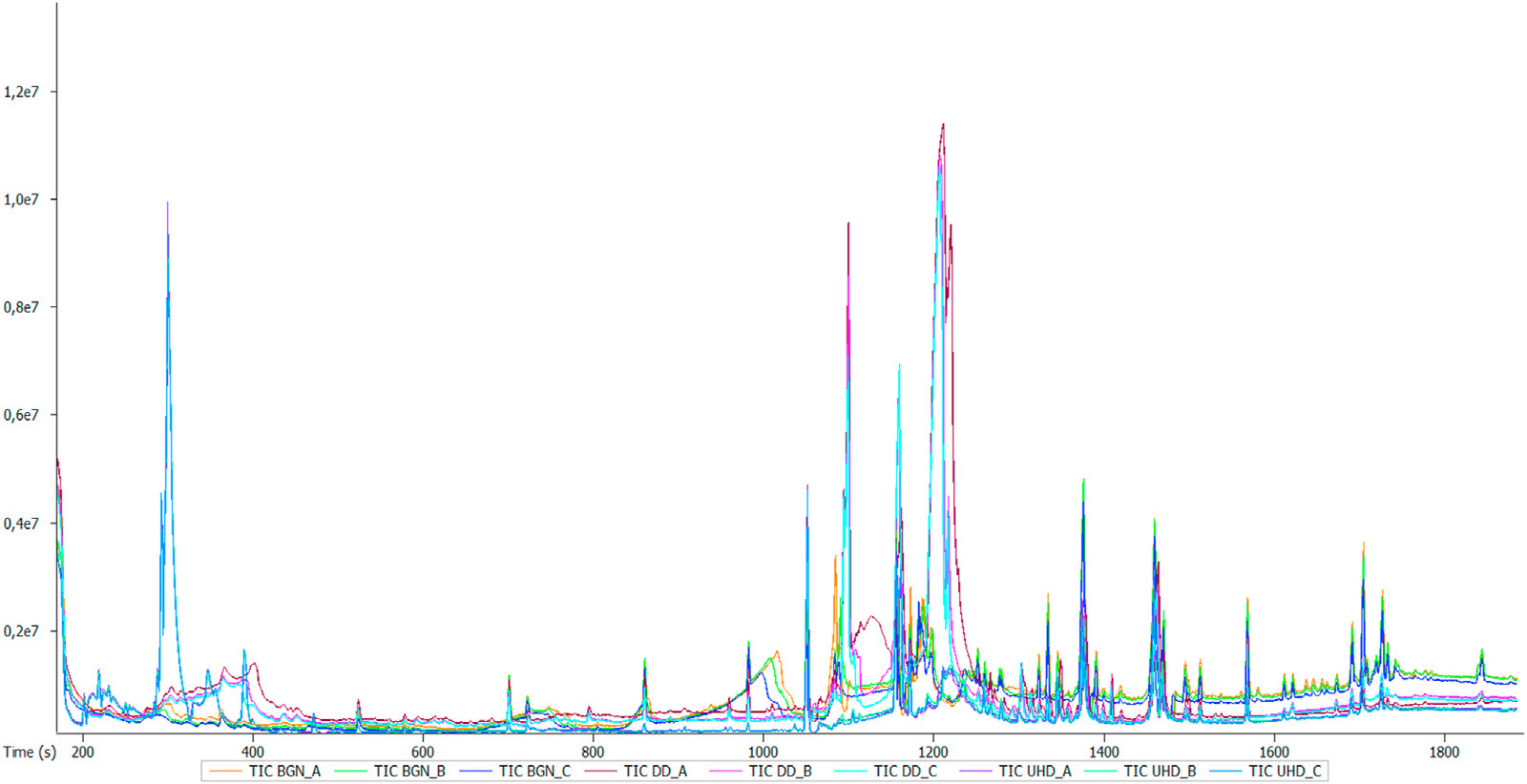
GC-HRTOF-MS chromatogram of the BGN (Bambara groundnut), DD (Dehulled *dawadawa*) and UHD (Unhulled *dawadawa*) samples.

**Figure 2:**
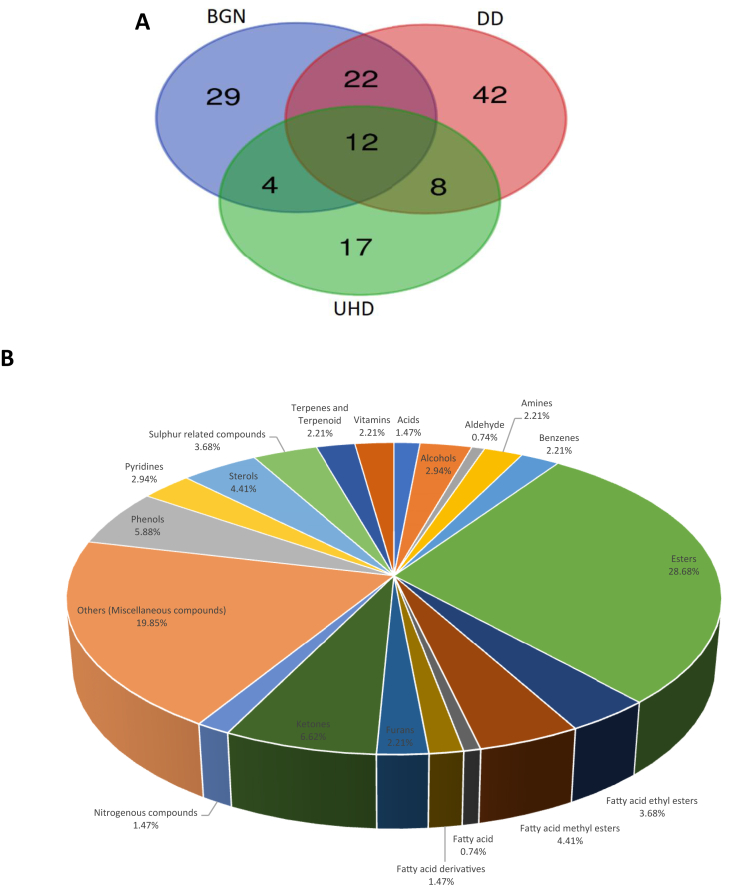
(A) Venn diagram showing the relationship between the metabolites in BGN (Bambara groundnut), DD (Dehulled *dawadawa*) and UHD (Unhulled *dawadawa*) samples, (B) Pie chart showing percentage distribution of the compounds.

The metabolic compounds of BGN and the two *dawadawa* produced (unhulled and dehulled) were analyzed using GC-HRTOF-MS. This is the first report to the best of our knowledge to profile and investigate the metabolites of BGN, unhulled (UHD) and dehulled (DD) *dawadawa* using GC-HRTOF-MS. In total, 134 metabolites were identified, and their identities presented in Table 1. Figure 1 represents the GC-HRTOF-MS chromatogram of BGN, DD and UHD samples. The group of compounds detected were terpenes and terpenoids (2%), amines (2%), sulphur related compounds (4%), ketones (7%), pyridines (3%), vitamins (2%), esters including fatty acid methyl and ethyl esters (37%), alcohols including sterols (7%), phenols (6%) and other miscellaneous compounds (20%). Eight compounds were identified in both *dawadawa* products, 29 in BGN, 42 in only DD, 17 in only UHD and 12 in all the samples analyzed (Figure 2A). Generally, more metabolites were detected in DD samples as compared to UHD, which might be attributed to increased microbial activity enhancing metabolic activities and better breakdown and/or formation of compounds. This was also the observation in an earlier study (Adebiyi et al., 2019; Adebiyi, 2020) and can be related to higher antioxidant activities and antinutritional factors (ANFs) in UHD as compared to DD samples, which might influence microbial activities. Some of the compounds identified in Table 1, were not detected in the raw BGN, but observed in DD and UHD samples. It can thus be speculated that these compounds were presumably produced during fermentation. The major metabolites that were only found in the fermented condiments include 9,12-octadecadienoic acid (Z,Z)-, 2-hydroxy-1-(hydroxymethyl)ethyl ester (5.13%), carbonic acid, 2-dimethylaminoethyl 2-methoxyethyl ester (7.3%), lauric acid, ethyl ester (10.2%), maltol (4%) and palmitic acid, ethyl ester (17.7%) for dehulled *dawadawa* and 2-methylbutyl propanoate (4.7%), 2-undecen-4-ol (4.7%), benzoic acid,4-amino-4-hydroximino-2,2,6,6-tetramethyl-1-piperidinyl ester (8.2%), ë-tocopherol (4.3%) and indoline, 2-(hydroxydiphenylmethyl)- (26.1%) for unhulled *dawadawa* samples (Table 1).

**Table 1:** Metabolites identified in Bambara groundnut and *dawadawa* (dehulled and unhulled) samples.

t_R_ (min)	Compound name and metabolite class	Observed *m/z*	*m/z* fragments	MF	Percentage peak areas		
					BGN	DD	UHD
	**Acids**						
04:47	6-Methylbicyclo[2.2.1]hept-2-ene-5-carboxylic acid	122.4808	65.9583, 105.1135	C_9_H_12_O_2_	ND	ND	4.69
06:27	1-Hydroxycyclohexanecarboxylic acid	131.6358	68.0502, 98.9424	C_7_H_12_O_3_	ND	ND	3.46
	**Alcohols**						
05:03	2-undecen-4-ol	192.9805	71.0490, 131.0703	C_11_H_22_O	ND	1.98	4.70
06:02	Maltol	126.0312	55.0180, 71.0128	C_6_H_6_O_3_	4.85	4.00	ND
06:03	Phenylethyl alcohol	122.0728	91.0544, 122.0728	C_8_H_10_O	ND	1.36	ND
17:00	1-Hexadecanol	196.2187	55.0544, 83.0856	C_16_H_34_O	0.10	ND	ND
	**Aldehyde**						
08:11	à-Ethylidenebenzeneacetaldehyde	146.0728	115.0544, 138.0913	C_10_H_10_O	ND	0.06	ND
	**Amines**						
14:13	1-Naphthalenamine, N-ethyl-	171.1045	129.0702, 156.0810	C_12_H_13_N	ND	0.12	ND
12:12	N-acetylphenethylamine	163.0994	30.0342, 104.0623	C_10_H_13_NO	ND	0.62	ND
13:20	p-Aminobiphenyl	169.0888	141.0700, 167.0733	C_12_H_11_N	ND	0.12	ND
	**Benzenes**						
07:53	Benzene, 1,3-bis(1,1-dimethylethyl)-	190.1711	124.0756, 175.1482	C_14_H_22_	ND	0.04	ND
10:13	Benzeneethanol, à-(phenylmethyl)-	208.2062	92.0622, 103.0544	C_15_H_16_O	ND	0.49	ND
24:55	Benzeneethanamine, 2-fluoro-á,3,4-trihydroxy-N-isopropyl-	226.2167	59.0367, 72.0445	C_11_H_16_FNO_3_	0.66	0.21	ND
	**Esters**						
03:55	Benzoic acid, 4-amino-, 4-hydroximino-2,2,6,6-tetramethyl-1-piperidinyl ester	120.4566	80.4620, 83.5630	C_16_H_23_N_3_O_3_	ND	ND	8.23
06:56	Benzofenac methyl ester	174.1069	61.0106, 91.0211	C_16_H_15_ClO_3_	0.05	ND	ND
07:08	Benzoic acid, 4-amino-, 4-acetoxy-2,2,6,6-tetramethyl-1-piperidinyl ester	153.0501	107.1252, 120.4566	C_18_H_26_N_2_O_4_	0.17	ND	1.23
08:27	Cyclobutanecarboxylic acid, 2-dimethylaminoethyl ester	151.1098	58.0653, 71.0730	C_9_H_17_NO_2_	ND	0.38	ND
09:18	Propanoic acid, 2-methyl-, 2,2-dimethyl-1-(1-methylethyl)-1,3-propanediyl ester	329.0325	43.0543, 71.0492	C_16_H_30_O_4_	0.34	0.25	ND
09:37	Propanoic acid, 2-methyl-, 3-hydroxy-2,2,4-trimethylpentyl ester	174.1206	71.0492, 89.0598	C_12_H_24_O_3_	0.33	ND	ND
10:19	Fumaric acid, ethyl 2,3,5-trichlorophenyl ester	167.1065	99.0442, 127.0390	C_12_H_9_Cl_3_O_4_	0.07	ND	0.57
10:34	Fumaric acid, monoamide, N,N-dimethyl-, 3-chlorophenyl ester	185.0676	98.0602, 126.0552	C_12_H_12_ClNO_3_	ND	0.16	ND
11:04	Phthalic acid, 3,4-dichlorophenyl methyl ester	194.0571	77.0386, 163.0392	C_15_H_10_Cl_2_O_4_	0.22	ND	ND
11:04	Phthalic acid, methyl 4-(2-phenylprop-2-yl)phenyl ester	283.0486	103.0139, 163.0307	C_24_H_22_O_4_	ND	0.10	0.33
11:08	4-Butylbenzoic acid, 2-dimethylaminoethyl ester	161.1200	58.0653, 71.0731	C_15_H_23_NO_2_	ND	0.19	ND
12:02	3,4-Dimethyl-2-(3-methyl-butyryl)-benzoic acid, methyl ester	208.5497	54.5083, 191.4042	C_15_H_20_O_3_	ND	ND	3.42
12:33	Ethyl 2-cyano-3-methylbutanoate	153.9684	68.0387, 82.5285	C_8_H_13_NO_2_	0.10	ND	0.61
12:57	6-Methoxythymyl 2-methylbutyrate	180.2455	121.4904, 165.0691	C_16_H_24_O_3_	ND	ND	0.42
13:23	Phthalic acid, monoamide, N-ethyl-N-(3-methylphenyl)-, ethyl ester	194.0570	149.0235, 177.0545	C_19_H_21_NO_3_	0.04	ND	ND
13:25	Butyric acid, thio-, S-hexyl ester	194.1546	73.0543, 71.0492	C_10_H_20_OS	ND	0.23	ND
15:42	Fumaric acid, butyl 2-phenylethyl ester	267.9997	104.0623, 203.0943	C_16_H_20_O_4_	ND	0.33	ND
16:43	Ethyl 13-methyl-tetradecanoate	270.2554	88.0520, 101.0599	C_17_H_34_O_2_	ND	0.17	ND
16:56	Phthalic acid, heptyl tridec-2-yn-1-yl ester	460.9532	57.0701, 149.0236	C_28_H_42_O_4_	0.42	ND	ND
17:45	Benzenepropanoic acid, 3,5-bis(1,1-dimethylethyl)-4-hydroxy-, methyl ester	292.2035	147.0808, 277.1799	C_18_H_28_O_3_	0.02	ND	ND
17:55	DL-Alanine, N-methyl-N-(byt-3-yn-1-yloxycarbonyl)-, tridecyl ester	224.1825	86.0966, 154.0738	C_22_H_39_NO_4_	ND	1.67	ND
17:57	Phthalic acid, 8-chlorooctyl nonyl ester	236.2140	148.8379, 205.4445	C_25_H_39_ClO_4_	0.56	0.94	0.30
17:57	Phthalic acid, 2-chloropropyl heptyl ester	224.0991	149.0235, 205.0860	C_18_H_25_ClO_4_	0.53	ND	0.28
17:58	Phthalic acid, 8-chlorooctyl decyl ester	224.1005	103.0392, 149.0235	C_26_H_41_ClO_4_	0.57	0.33	0.60
18:20	Fumaric acid, 2,6-dimethoxyphenyl dodec-2-en-1-yl ester	213.1026	68.0386, 153.9559	C_24_H_34_O_6_	ND	1.10	2.60
18:30	L-Proline, N-valeryl-, decyl ester	219.0079	55.1733, 84.0285	C_20_H_37_NO_3_	ND	0.86	ND
20:10	2-Methylbutyl propanoate	142.8595	56.5643, 70.0906	C_8_H_16_O_2_	ND	ND	4.68
21:00	Octanoic acid, 2-dimethylaminoethyl ester	218.0598	58.0652, 72.0808	C_12_H_25_NO_2_	2.48	1.98	ND
22:28	Carbonic acid, 2-dimethylaminoethyl 2-methoxyethyl ester	194.1912	58.0652, 71.0729	C_8_H_17_NO_4_	4.65	7.33	ND
23:11	Phthalic acid, dicyclohexyl ester	300.2082	149.0236, 167.0342	C_20_H_26_O_4_	0.47	0.11	0.82
24:06	Isophthalic acid, phenylethyl undecyl ester	267.0185	104.0825, 131.6355	C_27_H_36_O_4_	ND	0.02	0.46
24:19	9,12-Octadecadienoic acid (Z,Z)-, 2-hydroxy-1-(hydroxymethyl)ethyl ester	348.0901	67.0543, 262.2299	C_21_H_38_O_4_	5.82	5.14	ND
24:29	Octadecanoic acid, 2,3-dihydroxypropyl ester	359.3167	74.0362, 98.0728	C_21_H_42_O_4_	2.32	ND	ND
25:30	Butylphosphonic acid, decyl 4-(2-phenylprop-2-yl)phenyl ester	472.3099	221.1319, 457.2876	C_29_H_45_O_3_P	0.06	ND	ND
25:30	Succinic acid, 2-chloro-6-fluorophenyl phenethyl ester	400.9841	105.0699, 279.2308	C_18_H_16_ClFO_4_	ND	0.32	ND
25:37	Succinic acid, 3,4-dimethylphenyl 2-(dimethylamino)ethyl ester	312.3026	58.0135, 71.7611	C_16_H_23_NO_4_	0.37	0.16	ND
25:37	Carbonic acid, 2-dimethylaminoethyl isobutyl ester	186.1471	58.0652, 71.0729	C_9_H_19_NO_3_	0.21	0.27	ND
29:25	Urs-12-en-24-oic acid, 3-oxo-, methyl ester, (+)-	427.3893	189.1643, 218.2032	C_31_H_48_O_3_	0.14	ND	ND
30:39	Olean-12-en-28-oic acid, 3-oxo-, methyl ester	452.3664	203.1796, 262.1931	C_31_H_48_O_3_	0.74	ND	ND
	**Fatty acid ethyl esters**						
17:08	Pentadecanoic acid, ethyl ester	270.2552	88.0520, 101.0599	C_17_H_34_O_2_	ND	0.25	ND
18:00	9-hexadecenoic acid, ethyl ester	282.2556	69.0699, 88.0521	C_18_H_34_O_2_	ND	0.26	ND
18:14	Lauric acid, ethyl ester	228.2055	88.0521, 101.0600	C_14_H_28_O_2_	ND	10.15	ND
18:19	Palmitic acid, ethyl ester	285.2786	88.0522, 101.0601	C_18_H_36_O_2_	ND	17.74	ND
23:29	Stearic acid, ethyl ester	312.2990	88.0520, 101.0599	C_20_H_40_O_2_	ND	1.41	ND
	**Fatty acid methyl esters**						
15:59	Myristic acid, methyl ester	256.2399	88.0521, 101.0600	C_16_H_32_O_2_	ND	0.75	ND
17:30	Hexadecanoic acid, methyl ester	270.2556	74.0363, 87.0442	C_17_H_34_O_2_	6.98	ND	ND
19:15	9,12-Octadecadienoic acid, methyl ester	294.2561	81.0699, 95.0858	C_19_H_34_O_2_	2.55	ND	ND
19:19	trans-13-Octadecenoic acid, methyl ester	296.2714	55.0543, 74.0363	C_19_H_36_O_2_	0.73	ND	ND
19:31	Octadecanoic acid methyl ester	298.2871	74.0363, 143.1070	C_19_H_38_O_2_	1.61	ND	ND
23:00	Cerotic acid, methyl ester	356.3559	74.0363, 87.0442	C_27_H_54_O_2_	ND	1.41	ND
	**Fatty acid**						
18:05	Palmitic acid	256.2404	60.0207, 73.0284	C_16_H_32_O_2_	4.03	ND	ND
	**Fatty acid derivatives**						
20:29	Myristic acid amide	227.2204	59.0367, 72.0445	C_14_H_29_NO	ND	1.04	ND
22:55	2-monopalmitin	331.2852	104.0738, 128.5062	C_19_H_38_O_4_	7.88	3.65	ND
	**Furans**						
03:35	Furanoeudesma-1,4-diene	108.0683	47.0327, 64.0181	C_15_H_18_O	ND	1.50	ND
07:46	3-Butene-1,2-diol, 1-(2-furanyl)-	128.0357	49.0073, 97.0286	C_8_H_10_O_3_	2.39	1.90	0.32
19:45	Furfuryl ether	176.0922	81.0335, 143.0342	C_10_H_10_O_3_	9.31	ND	ND
	**Ketones**						
04:09	Hex-4-yn-3-one	95.8902	67.0060, 68.0471	C_6_H_8_O	ND	ND	1.02
06:02	3-Acetoxy-2-methyl-pyran-4-one	129.0913	71.0128, 126.0312	C_8_H_8_O_4_	3.57	3.67	ND
07:41	2-Coumaranone	134.0364	78.0464, 106.0414	C_8_H_6_O_2_	ND	0.12	ND
08:44	Ethanone, 1-(2-hydroxy-5-methylphenyl)-	150.0677	107.0493, 135.0442	C_9_H_10_O_2_	0.44	0.30	ND
09:47	7-Chloro-1,3,4,10-tetrahydro-10-hydroxy-1-[[2-[1-pyrrolidinyl]ethyl]imino]-3-[3-(trifluoromethyl)phenyl]-9(2H)-acridinone	268.9973	84.0809, 132.0548	C_26_H_25_ClF_3_N_3_O_2_	0.91	0.15	3.75
11:04	2-(6-Chloro-3-nitro-4-phenyl-quinolin-2-ylsulfanyl)-1-(2,3-dihydro-benzo[1,4]dioxin-6-yl)- ethanone	194.0574	132.5996, 163.0307	C_25_H_17_ClN_2_O_5_S	ND	0.12	0.36
17:32	7,9-Di-tert-butyl-1-oxaspiro(4,5)deca-6,9-diene-2,8-dione	276.1718	175.119, 205.0861	C_17_H_24_O_3_	0.11	ND	ND
23:20	2-methoxy-,2-octen-4-one,	152.0474	99.0443, 114.0677	C_9_H_16_O_2_	0.19	0.08	ND
28:04	3,6,13,16-tetraoxatricyclo[16.2.2.2(8,11)]tetracosa-8,10,18,20,21,23-hexaene-2,7,12,17-tetrone	380.0489	208.0519, 341.0657	C_20_H_16_O_8_	0.08	ND	ND
	**Nitrogenous compounds**						
04:48	Indoline, 2-(hydroxydiphenylmethyl)-	314.5135	103.0505, 118.4013	C_21_H_19_NO	ND	ND	26.11
07:34	Indole, 3-(2-(diethylamino)ethyl)-	130.6049	85.5811, 129.5840	C_14_H_20_N_2_	ND	ND	0.16
	**Others (Miscellaneous compounds)**						
03:45	N-[3,3′-dimethoxy-4'-(2-piperidin-1-yl-acetylamino)-biphenyl-4-yl]-2-piperidin-1- yl-acetamide	128.0471	93.0701, 98.0364	C_28_H_38_N_4_O_4_	0.55	5.14	ND
05:50	Succinic anhydride	102.0283	36.5607, 55.5498	C_4_H_4_O_3_	ND	ND	6.71
06:22	Decamethylcyclopentasiloxane	358.0680	73.0469, 266.9992	C_10_H_30_O_5_Si_5_	0.12	0.05	ND
06:27	N,N-Dimethylglycine	103.0631	42.0338, 58.0653	C_4_H_9_NO_2_	ND	0.62	ND
06:39	1H-Imidazole-4-methanol	98.0364	69.0335, 97.0286	C_4_H_6_N_2_O	ND	2.31	ND
06:52	Thiourea, N-(3-methyl-2-pyridinyl)-N'-[(tetrahydro-2-furanyl)methyl]-	332.0663	44.0733, 150.0677	C_12_H_17_N_3_OS	ND	0.12	ND
07:32	Catecholborane	120.0570	100.0759, 148.0994	C_6_H_5_BO_2_	ND	0.71	ND
07:42	2-Benzoxazolamine, N-(1,1-dimethylethyl)-	153.9813	105.0764, 133.6238	C_11_H_14_N_2_O	ND	ND	0.32
08:43	Dodecamethylcyclohexasiloxane	434.0840	73.0468, 341.0179	C_12_H_36_O_6_Si_6_	0.76	0.91	1.69
08:44	Phenyl-1,2-diamine, N,4,5-trimethyl-	149.8967	106.1084, 134.6487	C_9_H_14_N_2_	ND	ND	0.36
09:53	Benzaldehyde, 3-methoxy-4-[(2-methylphenyl)methoxy]-	195.1248	105.0701, 132.0810	C_16_H_16_O_3_	ND	1.00	ND
11:15	4,4′-Dichlorodibutyl ether	158.0202	91.0312, 93.0280	C_8_H_16_Cl_2_O	0.03	ND	ND
11:40	Tetradecamethylcycloheptasiloxane	508.1064	73.0467, 281.0513	C_14_H_42_O_7_Si_7_	2.04	1.72	ND
11:50	Tetradonium Bromide	165.0703	58.0653	C_17_H_38_BrN	0.13	ND	ND
12:58	2,3,5,6-Tetrafluoroanisole	180.0782	137.0569, 165.0548	C_7_H_4_F_4_O	ND	0.23	ND
13:14	3-Methyl-4-phenyl-1H-pyrrole	157.0886	127.5468, 155.9884	C_11_H_11_N	ND	1.19	1.12
13:24	2-propynenitrile, 3-fluoro-	69.0591	53.4495, 81.5012	C_3_FN	ND	ND	1.05
14:20	Hexadecamethyl-cyclooctasiloxane	580.1260	73.0468, 355.0702	C_16_H_48_O_8_Si_8_	1.71	1.41	1.53
17:09	2,7-Dimethylcarbazole	195.1039	140.0702, 167.0726	C_14_H_13_N	ND	0.03	ND
17:50	1-Methyl-2,5-dipropyldecahydroquinoline	195.4010	86.6185, 166.1360	C_16_H_31_N	ND	ND	0.15
19:41	2-(1-Pyrrolidinyl)ethyl 4-propoxysalicylate	238.7279	83.5285, 96.8999	C_16_H_23_NO_4_	ND	ND	0.43
19:42	Monoethanolamine stearic acid amide	282.2785	85.0523, 98.0602	C_20_H_41_NO_2_	3.21	ND	ND
20:47	3-Cyclopentylpropionamide, N,N-dimethyl-	170.1547	45.0574, 87.0680	C_10_H_19_NO	0.72	0.12	ND
22:07	Pyrrolo[1,2-a]pyrazine-1,4-dione, hexahydro-3-(phenylmethyl)-	244.1207	125.0709, 153.0660	C_14_H_16_N_2_O_2_	ND	0.22	ND
22:25	Bis(2-(Dimethylamino)ethyl) ether	156.1014	58.0652, 71.0729	C_8_H_20_N_2_O	7.95	0.25	ND
24:55	Acetaldehyde, diethylhydrazone	114.0678	71.0492, 99.0443	C_6_H_14_N_2_	ND	0.09	ND
26:59	S-[2-[N,N-Dimethylamino]ethyl]N,N-dimethylcarbamoyl thiocarbohydroximate	218.0808	58.0652, 71.0729	C_8_H_17_N_3_O_2_S	0.20	ND	ND
	**Phenols**						
09:14	2-methyl-6-tert-butylphenol	164.1196	121.0649, 149.0963	C_11_H_16_O	0.02	ND	ND
12:02	2,4-Di-tert-butylphenol	206.1665	57.0700, 191.1431	C_14_H_22_O	2.00	ND	ND
12:04	Phenol, 2,4,6-tris(1-methylethyl)-	220.1824	177.1274, 205.1588	C_15_H_24_O	0.13	0.13	0.56
12:05	Butylated Hydroxytoluene	220.1824	43.0179, 205.1589	C_15_H_24_O	0.19	0.12	0.65
12:57	2-tert-Butyl-4-methoxyphenol	180.0781	137.0596, 165.0548	C_11_H_16_O_2_	0.35	ND	ND
16:48	Resorcinol/3-Hydroxyphenol	110.0602	82.0289, 201.1147	C_6_H_6_O_2_	ND	0.15	ND
16:52	Taxicatigenin	153.9559	68.0387, 124.5019	C_8_H_10_O_3_	ND	ND	0.58
22:14	Phenol, 2,2′-methylenebis[6-(1,1-dimethylethyl)-4-methyl-	340.2401	161.0964, 177.1277	C_23_H_32_O_2_	3.52	0.76	5.21
	**Pyridines**						
11:18	o-phenylpyridine	154.9679	126.5207, 155.9841	C_11_H_9_N	ND	ND	0.29
11:19	m-Phenylpyridine	155.0730	127.0544, 156.0764	C_11_H_9_N	ND	0.46	0.23
15:05	4-pyridinamine, N-[(4-methoxyphenyl)methylene]-	212.1306	91.0544, 197.1073	C_13_H_12_N_2_O	ND	0.14	ND
20:56	2,6-diphenyl-pyridine,	231.1044	58.0653, 202.0777	C_17_H_13_N	ND	0.16	ND
	**Sterols**						
28:08	Ergosta-5,24-dien-3-ol, (3á)-	384.3342	281.2269, 314.2608	C_28_H_46_O	0.04	ND	ND
28:11	Campesterol	400.3701	145.1014, 213.1642	C_28_H_48_O	0.49	0.12	ND
28:24	Stigmasterol	412.3710	83.0856, 159.1172	C_29_H_48_O	1.39	0.41	ND
28:53	Stigmasta-5,24(28)-dien-3-ol, (3á,24Z)-	412.3703	314.2609, 281.2269	C_29_H_48_O	0.51	0.15	ND
29:02	Cycloeucalenol	412.3684	95.0857, 107.0858	C_30_H_50_O	0.17	ND	ND
29:06	Olean-12-en-3-ol	426.3862	203.1797, 218.2032	C_30_H_50_O	0.29	ND	ND
	**Sulphur related compounds**						
04:13	Dimethyl trisulfide	125.9627	78.9671, 127.9585	C_2_H_6_S_3_	ND	1.88	ND
07:16	2,5-dihydrothiopene	86.0364	45.0336, 57.0336	C_4_H_6_S	ND	1.52	ND
08:18	Hemineurine	143.0401	85.0108, 112.0217	C_6_H_9_NOS	ND	0.10	ND
20:45	O-Ethyl S-2-diethylaminoethyl ethylphosphonothiolate	257.7809	85.6057, 98.9677	C_10_H_24_NO_2_PS	ND	ND	1.21
21:12	1H-Indole-3-carbonitrile, 2-(4-chlorobenzenesulfonylmethyl)-1-methyl-	225.1118	169.1224, 201.1485	C_17_H_13_ClN_2_O_2_S	ND	0.08	ND
	**Terpenes and Terpenoid**						
04:56	Eucalyptol	154.1354	81.0700, 93.0701	C_10_H_18_O	0.26	ND	ND
07:02	Naphthalene	128.0622	76.0307, 99.0442	C_10_H_8_	0.34	0.27	ND
28:47	Clionasterol	414.3864	145.1015, 213.1643	C_29_H_50_O	0.61	0.15	ND
	**Vitamins**						
26:08	ë-Tocopherol	402.3499	137.0599, 177.0914	C_27_H_46_O_2_	3.43	1.05	4.30
26:51	ç-Tocopherol	416.3654	151.0755, 191.1069	C_28_H_48_O_2_	1.77	0.41	3.28
23:58	dl-7-azatryptophan	204.0760	88.0336, 131.0524	C_10_H_11_N_3_O_2_	ND	0.28	1.22

t_R_ – Retention time; *m/z* – mass-to-charge ratio; MF – Molecular formula; ND- Not detected; BGN – Bambara groundnut, DD – Dehulled *dawadawa*; UHD – Unhulled *dawadawa*

Esters were the principal compounds reported in this study. Other similar studies on fermented condiments contradict this observation, with pyrazines being the major constituent in *sonru*, *afitin*, *iru* (Azokpota et al., 2008), acids the dominant group in castor oil bean fermented condiment (Ojinnaka and Ojimelukwe, 2013), aldehydes in locust bean *daddawa*, soybean and melon seed *ogiri* (Onyenekwe et al., 2012), while aldehydes, acids and ketones were reported to dominate *dawadawa* from BGN using *Bacillus* species (Akanni et al., 2018a). Nevertheless, esters are major metabolite groups common to several fermented condiments in Africa and are mostly formed during fermentation by esterification of alcohols with fatty acids (Fan and Qian, 2005). Chemical reactions between alcoholic metabolites as well as microbial acidic metabolites could also lead to the formation of esters during fermentation. Their contributions to food aroma/odour are important, combined with the fact that esters at ambient temperatures are highly volatile and their perception thresholds are much lower compared to their alcohol precursors (Nogueira et al., 2005). Compounds belonging to the esters group constitutes 29% (Figure 2B) of the total metabolites recorded and were more prominent in the DD as compared to UHD. Phthalic acid 8-chlorooctyl decyl ester, phthalic acid dicyclohexyl ester and phthalic acid 8-chlorooctyl nonyl ester were the major esters detected in BGN, DD and UHD (Table 1).

The identified compounds in this study could be as a result of the breakdown and constituents in BGN such as proteins, lipids and other bioavailable compounds through the activities of the microbial enzymes. As reflected in the GC-HRTOF-MS data presented in Table 1, fermentation of BGN into derived *dawadawa* led to the formation, increase, as well as decrease of some compounds. Formation of these constituents could be attributed to the presence of microorganisms involved in the fermentation process and other processing factors as well as operations involved during *dawadawa* preparation (Azokpota et al., 2010). Compounds belonging to an acid group were only present in UHD samples, which can be attributed to the relatively longer fermentation period for the UHD samples. Acids are sometimes considered as undesirable compounds that confer unpleasant characteristics such as rancid, sweaty and pungent flavors (Frauendorfer and Schieberle, 2008), although they have been reported to confer some acidic, fruity and sour notes in fermented foods (Park et al., 2013).

Compounds belonging to the sulphur related group were mostly present in the DD with none detected in the BGN, in agreement with the study of Akanni et al. (2018b), in which sulphur-related compounds were equally not detected in the raw BGN. Dimethyl trisulfide (sulphur related compound) is known to confer meaty, sulfureous, eggy, alliaceous, cooked, savory, and onion note (Liu et al., 2012). It can also be identified as a possible product of amino acid metabolism (Tamman et al., 2000). Speculated possible amino acid degradation and significantly (p ≤ 0.05) different values in the amino acid of BGN and derived *dawadawa* (Adebiyi et al., 2019) could also explain the detected amine-related and nitrogenous compounds (Table 1).

Both ketones and aldehydes are formed by fatty acids beta-oxidation as well as oxidation catalyzed by lipoxygenase and hyper-oxidase enzymes, yielding important flavor compounds (Nzigamasabo, 2012). Aldehydes are not only flavor components, but also known as vital reactants associated with heterocyclic compounds formation (Ziegleder, 2009). Ketones are generally derived from amino acid and lipid degradation with the presence of these compounds having an impact on food flavor (Adebo et al., 2018). Nine ketones were detected, *i.e*. six from DD and three from UHD. The ketones in UHD decreased, as compared to the raw BGN, whereas there was a slight increase of the ketone group in DD (relative to the percentage peak areas). Dehulling of the seedcoats exposed fats related components to more oxygen coupled with removal of available antioxidants in the hull. This thus suggests that fat oxidation would likely be higher in the dehulled samples as compared to the unhulled samples (Akkad et al., 2019). Therefore, an increased level of ketones in the dehulled samples might be due to partial oxidation of the alcohols as well as synthesis through several metabolic pathways, particularly reduction of methyl-ketone (Curioni et al., 2002; Akkad et al., 2019). This may be associated with the disappearance and/or reduction of some ketones in BGN and *dawadawa*.

Alcohols constituted 3% of metabolites in Figure 2B and are generated by reduction reaction of corresponding aldehydes and oxidation of acids (Pham et al., 2008). According to Estrella et al. (2004), aldehydes and ketones are relatively unstable intermediate compounds and can easily be reduced to alcohols. In total, four alcohols were detected in this study, *i.e*. 2-undecen-4-ol at high levels in two fermented samples, maltol in BGN and DD, phenylethyl alcohol in DD, with 1-hexadecanol in BGN. The phenylethyl alcohol compound has a rose-like odor and is known as one of the major Korean fermented soy sauce odor-active compounds (Lee et al., 2006), suggesting that these alcohol-related compounds might contribute to the flavor of these *dawadawa* samples. A decrease in the number of alcohols in the fermented samples might be due to the heat treatment (i.e. cooking) applied during processing (Cho et al., 2017; Wang et al., 2019).

The pyridines group was not detected in the BGN except in the *dawadawa* samples, indicative of a formation of these compounds. Pyridines are usually formed during cooking of food (Gupta et al., 2019) or meat, probably due to the reaction of amino acids with alkanals (Hui, 2012). They are classified as the flavor component of beer and as important organoleptic compounds of foods from cocoa, peanuts, cheeses, beans and barley (Maga, 1981). Due to the physical properties of BGN (hard to cook phenomenon), the seeds are usually cooked briefly then dehulled (depending on the product) prior to fermentation, which might explain the occurrence of pyridines in this study.

Three vitamin-related compounds (Table 1) were detected in *dawadawa* samples except for dl-7-azatryptophan, which was completely absent in BGN. Other notable vitamins observed were ç-Tocopherol and ë-Tocopherol, which are forms of vitamin E. Not only is vitamin E of nutritional and dietary importance, but it also functions as an antioxidant by preventing the propagation of lipid peroxidation (Frei, 2004). It was observed that in dl-7-azatryptophan, the peak area of UHD (1.22%) was higher than that of DD (0.28%), while UHD has the highest percentage peak area in all the vitamins reported. Furans are heterocyclic compounds, known to possess sweet, roasted, burnt, caramel and sugar notes as previously reported in *dawadawa* (Akanni et al., 2018a; Azokpota et al., 2008).

Phenols are a major group of antioxidants and of great significance due to their biological and free radical scavenging activities (Koleva et al., 2018). Compounds belonging to the phenol group were also identified in this study. There was formation of taxicatigenin, which is also known as 3,5-dimethoxyphenol in UHD sample. 3,5-dimethoxyphenol belongs to methoxyphenols (a class of compounds comprising of a methoxy group) and connected to the benzene ring of a phenol moiety. The occurrence of this compound and its presence in only UHD could further explain its higher antioxidant activity in a previous study (Adebiyi, 2020), as taxicatigenin is a bioactive compound with potential antioxidant activity (Nithya et al., 2018). Bioactive compounds are also known to inhibit microbial growth that might have contributed to lesser microbial activity in UHD samples, resulting in reduced pH values (Adebiyi et al., 2020). Compounds belonging to the sterols group were common in raw BGN but none of these sterols were detected in UHD. There are claims that naturally occurring plant cholesterols may promote the health of animals and humans once consumed regularly either as food supplements or naturally in foods for a reasonable amount of time (Ogbe et al., 2015).

Fatty acid methyl esters were common in raw BGN, with the formation of methyl esters (myristic acid and cerotic acid methyl esters) in DD, while none of the fatty acid methyl esters were detected in UHD. This difference might have been due to the cooking process adopted (i.e., boiling), as heat treatment is known to affect fatty acid constituents of foods (Ouazib et al., 2015). Hexadecanoic acid methyl ester and octadecanoic acid methyl ester were detected only in raw BGN and are both known as the most abundant saturated fatty acids in nature, reported in plants, animals, lower organisms and functions in cells as specific proteolipids (*i.e.* connected to internal cysteine residues through thioester bonds) (Anonymous, 2013).

## 4 Conclusion

A total of 134 metabolites were detected in Bambara groundnuts and derived *dawadawa* samples using GC-HRTOF-MS. From the two fermented samples, dehulled samples had the highest number of metabolites as compared to their unhulled counterparts. Compounds identified included esters, ketones, phenols, flavor related compounds and constituents that could confer organoleptic properties, nutritional and functional benefits of BGN and derived *dawadawa*. The BGN seeds and dehulled *dawadawa* possess beneficial components that can potentially be incorporated into human diets for health benefits. Further investigations into the quantification of the metabolites in this study are still needed, particularly for those significant metabolites obtained in all three samples. These would not only provide a better understanding of legume fermentation, but also assist in providing an insight into these significant metabolites that could potentially be biomarkers of *dawadawa*.

## Declarations

### Author contribution statement

Janet Adeyinka Adebiyi: Conceived and designed the experiments; Performed the experiments; Wrote the paper.

Patrick Berka Njobeh, Eugenie Kayitesi: Conceived and designed the experiments; Contributed reagents, materials, analysis tools or data.

Oluwafemi Ayodeji Adebo: Performed the experiments; Analyzed and interpreted the data.

### Funding statement

This work was supported by the 10.13039/501100001321National Research Foundation (NRF) of South Africa (120751) and the NRF of South Africa National Equipment Programme (99047).

### Data availability statement

Data included in article/supplementary material/referenced in article.

### Declaration of interests statement

The authors declare no conflict of interest.

### Additional information

No additional information is available for this paper.
